# ZooMonitor Community: A Tool for Evaluating Population-Level Behavior Questions

**DOI:** 10.3390/ani16121822

**Published:** 2026-06-12

**Authors:** Jason D. Wark, Nancy Kim Pham, John E. Andrews

**Affiliations:** 1 Animal Welfare Science Program, Lincoln Park Zoo, Chicago, IL 60614, USA; 2 South-East Zoo Alliance for Reproduction & Conservation, Yulee, FL 32097, USA; nancy.pham@sezarc.com; 3 AZA Population Management Center, Lincoln Park Zoo, Chicago, IL 60614, USA; jandrews@lpzoo.org

**Keywords:** animal behavior, multi-institutional, zoo, aquarium, collaboration

## Abstract

From plains zebras to zebra sharks, modern, accredited zoos and aquariums cooperate on the breeding and transfer of individuals from diverse species with unique needs. As a result, the housing and husbandry of a species varies across organizations. The degree to which these practices align with a species’ needs can influence well-being and reproduction. As a key measure of well-being, animal behavior is often the most practical and useful tool for evaluating the impact of housing and husbandry on animals. However, studies conducted at a single organization can be limited in their ability to explore these factors and a multi-institutional approach is needed. Recently, Lincoln Park Zoo launched the ZooMonitor Community, an expansion of the ZooMonitor behavior recording platform. This new collaboration tool is freely available to all zoos and aquariums to facilitate multi-institutional data sharing and project management. Here, we provide an overview of the ZooMonitor Community tool and highlight several recent Community projects on diverse taxa, including giraffes, Asian small-clawed otters, common seadragons, and southern ground hornbills. We hope the ZooMonitor Community can provide population managers with an accessible tool to address broad, species-level questions on care, well-being, and reproduction that can enhance the lives of animals in managed settings.

## Simple Summary

Animals living in modern, accredited zoos and aquariums are cooperatively managed across hundreds of organizations. Naturally, how animals are cared for can vary between organizations, from the size of habitats, the number of social partners, the types of food provided, and much more. Understanding how these factors can influence animal well-being and reproduction is of critical importance for zoos and aquariums as they seek to identify best (or better) practices. Comparing animal behavior across organizations has been the primary way to evaluate these factors but can be challenging with existing tools. To address this need, Lincoln Park Zoo launched the ZooMonitor Community, an expansion of the freely available ZooMonitor behavior data collection platform. The ZooMonitor Community introduces new ways to collaborate and share data across zoos and aquariums. Built-in features simplify and streamline project management. We provide a detailed overview of the ZooMonitor Community tool and highlight several recent Community projects on diverse taxa, including giraffes, Asian small-clawed otters, common seadragons, and southern ground hornbills. We hope the ZooMonitor Community can provide population managers with an accessible tool to address broad, species-level questions on care, well-being, and reproduction that can enhance the lives of animals in managed settings.

## 1. Introduction

The breeding and transfer of animals within accredited zoos and aquariums are cooperatively managed by specialized, expert groups. Within the Association of Zoos and Aquariums (AZA), this consists of Species Survival Plans^®^ (SSPs) tasked with coordinating population management. Historically, many of these groups have made their breeding and transfer recommendations primarily on the basis of genetics and demography. Although animal behavior may have been considered previously, recommendations are now increasingly incorporating behavior in a more explicit and systematic fashion. For example, within the Gorilla SSP, the formation of bachelor male groupings for non-breeding males was based on behavior research of the social compatibility of these all-male groupings [1,2]. In addition, as these population management groups represent species experts, their remit has extended beyond cooperative breeding purposes and recommendations are often sought on behavioral management for welfare purposes. This can include managing social introductions and group dynamics, recommendations for diets and feeding practices, and approaches for mediating abnormal behaviors, to name a few. Recent efforts have worked to formally document this collective knowledge through care guides that describe current best practices (e.g., Association of Zoos and Aquariums Animal Care Manuals [3]; European Association of Zoos and Aquaria (EAZA) Best Practice Guidelines [4]).

For many species, the foundation of animal behavior knowledge drawn upon by population management groups has been largely built through years of personal experience in caring for those species and guided by the species’ natural history. This accumulated professional wisdom is the backbone of animal care practices in modern, accredited zoos. Although valuable, this experience-based style of learning can be slow and, in some cases, may result in myths and folklore in care practices [5,6,7].

Complimenting this approach is the structured, scientific study of animal behavior in zoos and aquariums [8]. However, this approach is not without its own limitations. This knowledge base has been biased towards mammals and birds [6,9,10,11], although growing attention towards understudied taxa has begun to address this gap [12]. An additional challenge is the scale of zoo and aquarium research, as studies are often conducted with a small number of individuals at a single facility. As a consequence, findings from small sample size research are often limited in their ability to generalize to other individuals at different facilities [13]. Although these types of case studies still hold value in demonstrating successful (or unsuccessful) approaches from a methodological standpoint, they can fall short at advancing our knowledge of best care practices for a species.

Single-institution studies can also be limited in the questions they can address. Although data from a single institution can provide valuable insights, particularly for those dimensions of care that can be experimentally altered (e.g., diets, [14]; enrichment, [15,16]), some aspects of care cannot be easily modified at a single organization. For example, the effect of habitat size is of great interest to zoos and aquariums and has frequently been targeted by those opposed to zoos [17,18]. For smaller species, it may be possible to evaluate habitat size within a single facility through the use of experimental enclosures [19,20,21]. However, for megafauna, experimentally assessing habitat size at a single organization is typically not feasible, given the amount of space needed and requisite safety considerations (although not impossible, as demonstrated by [22]). Similarly, determining appropriate social group sizes can be challenging, given the limited number of individuals that are often housed within a single organization. To address these broader dimensions of care, powerful, multi-institutional research studies are needed [23,24,25]. These studies can support population management groups and draw from their expert knowledge base to advance our collective understanding of how to best care for species.

To facilitate collaborative research across zoos and aquariums, Lincoln Park Zoo created the ZooMonitor Community, an expansion of the ZooMonitor behavior data collection platform. This free tool brings new, accessible ways to create and manage multi-institutional research studies. To our knowledge, the ZooMonitor Community is the only tool available within the zoological community purpose-built to facilitate collaborative behavior data collection on any species. Herein, we describe this software and the steps required to initiate a multi-institutional study. We also share several case studies highlighting the potential of the ZooMonitor Community for use with diverse taxa and topics. We hope this introduction will encourage population managers to facilitate or lead multi-institutional research on species they manage.

## 2. Materials and Methods

### 2.1. Background

ZooMonitor (v. 5.0) is a freely available web application developed by Lincoln Park Zoo and built by Tracks Data Solutions for recording the behavior and space use of animals [26]. Launched in 2016, the ZooMonitor app has become a common behavior data collection tool within zoos and aquariums around the world, with accounts by more than 700 zoos and aquariums worldwide. ZooMonitor has been used to evaluate a myriad of species and topics in zoos. Some examples include space use and territoriality in Nile crocodiles [27], the impact of loose substrate on naked mole rats [28], the behavior of goats in an animal–visitor interaction program [29], and the comparison of musth in zoo-housed and free-ranging Asian elephants [30], to name a few.

**Figure 1:**
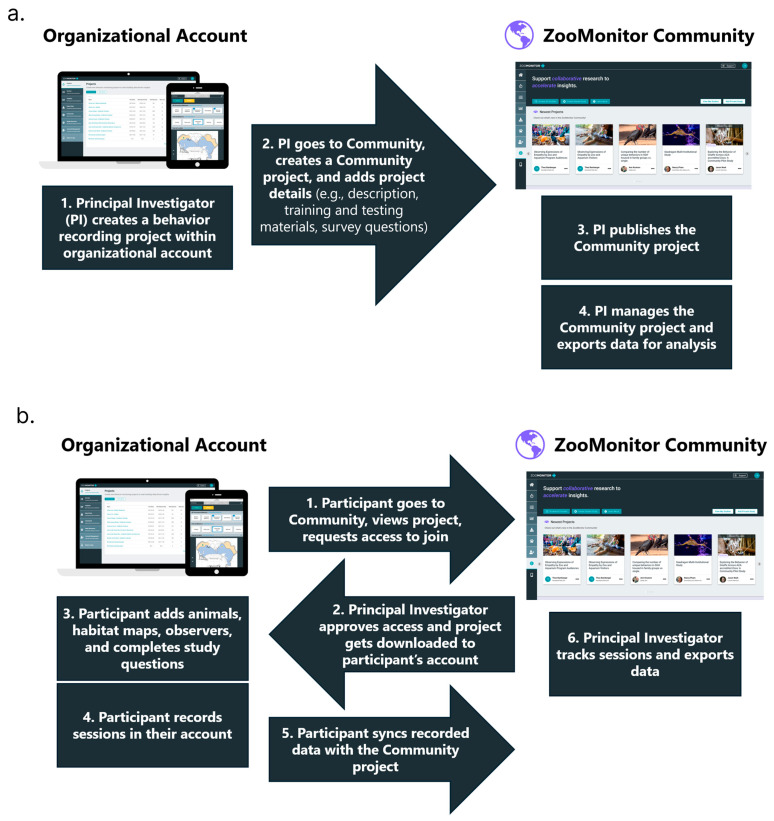
Steps for (**a**) Principal Investigators creating a shared project in the ZooMonitor Community, and for (**b**) participants to join and contribute to a shared Community project. See Figure 5 for a detailed view of the ZooMonitor Community interface.

Within ZooMonitor, all behavior data are privately stored within an organization’s database and, by default, are only accessible to organizational administrators. To facilitate the collaborative sharing of ZooMonitor data across organizational accounts, Lincoln Park Zoo created the ZooMonitor Community, an expansion of the ZooMonitor platform. The ZooMonitor Community is freely available to all ZooMonitor users (e.g., zoos, aquariums, sanctuaries, universities, museums) through their organization’s admin area. Using the ZooMonitor Community, researchers can create and share multi-institutional research studies through the ZooMonitor platform with collaborators around the world (Figure 1).

### 2.2. Project Creation

**Figure 2:**
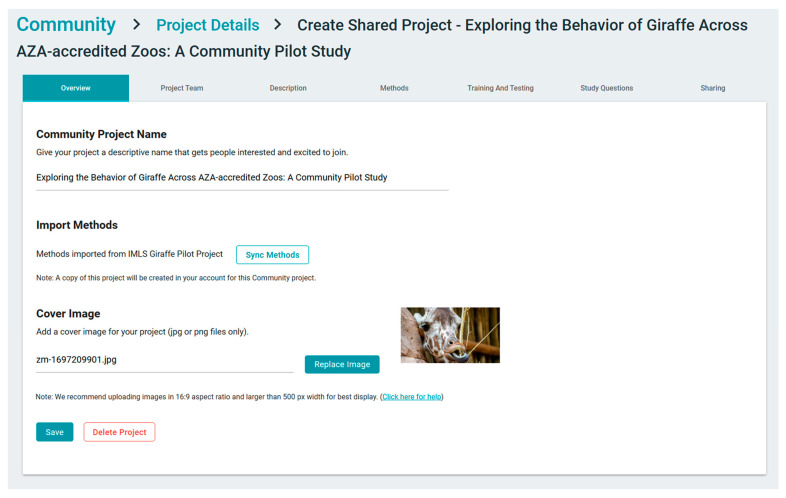
Screenshot of the Overview Tab when creating a study in the ZooMonitor Community.

When creating a new shared project within the ZooMonitor Community, the Principal Investigator (PI) adds important project details in a series of steps (Figure 1a). First, PIs select an existing project within their organizational account to import sampling methods and any questions on independent session variables (e.g., temperature, weather, crowd size; Figure 2). This allows the PI to test and refine the data collection protocol within their account before creating the Community project. After this step, the PI can then add additional information such as an abstract and descriptions of the data collection, reliability testing, and analysis procedures. As many multi-institutional research projects would need to be reviewed by an organizational research committee, the PI can also add a research proposal and other materials needed for review, such as an Institutional Animal Care and Use Committee (IACUC) or Institutional Review Board (IRB) approval letter. For projects that are supported by a zoo-related group (e.g., SSP or Taxon Advisory Group (TAG)), the PI can include an endorsement letter, which adds an “endorsed” label to the project in the Community.

In addition to session-based questions that are associated with the data collection project, PIs can include additional questions necessary for multi-institutional research. Organization-related questions (e.g., organization size, specific husbandry practices, habitat details, etc.) are completed by interested participants when requesting access to join the project. For research projects that are evaluating a specific housing or husbandry condition (e.g., solitary vs. social housing), these questions can be useful in screening participants when granting access to the project. Questions can also be added to gather animal-related (e.g., sex, age, rearing type) and habitat-related factors (e.g., habitat size, substrate type, indoor vs. outdoor). These questions are linked to the individual animals and habitats the participants add to their study in ZooMonitor.

### 2.3. Project Sharing

When all details have been added, the PI can publish the project to the Community, allowing ZooMonitor users at other organizations to view the project and request access to join the study. Community projects can be published in one of three ways: (a) Public, which allows any ZooMonitor user to view and request access to join the project; (b) Accredited, which permits only organizations within specific, regional accreditation groups (e.g., AZA-only), as specified by the PI, to view the project and request access; and (c) Private, which hides the project in the Community by default and provides the PI with a private code that can be shared with colleagues to allow them to view the project and request access to join. In addition, the PI can also specify whether data shared by participants is identifiable, which would preserve all identifying fields in the dataset (i.e., organization name, animal names, habitat names, and observer names), or anonymous, which pseudonymizes all identifiers.

After a participant is granted access to the Community study, a new blank copy of the project (i.e., with no existing data, animals, habitat maps, or observers) is created in their organizational account for data collection on the shared Community project (Figure 1b). Data recorded by those participants are first uploaded from tablet devices to the participants organizational account and then are manually synced to the shared Community project database by a project lead at each participating organization (i.e., the ZooMonitor user that requested access to join the study). This manual data syncing gives participants control over their data and allows them to make any necessary edits to their data before sharing with the PI.

### 2.4. Project Management

**Figure 3:**
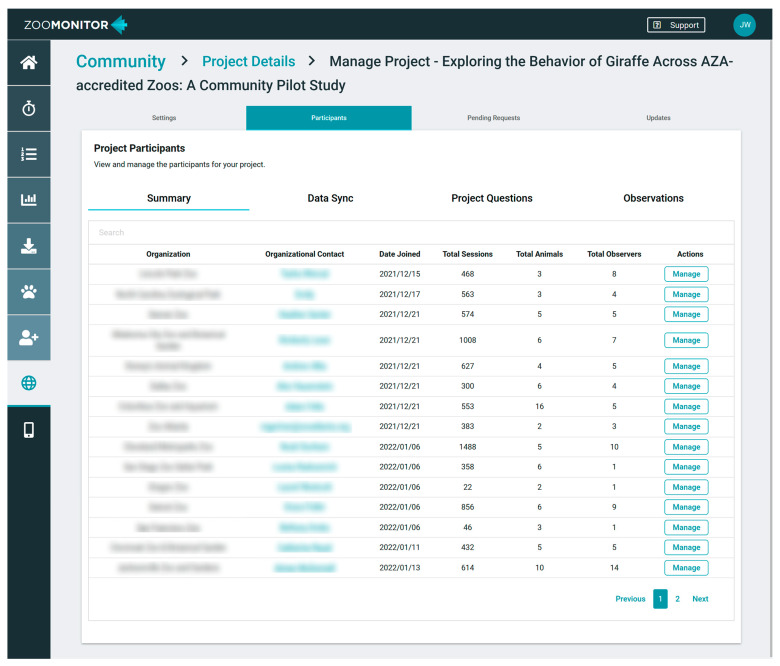
ZooMonitor Community screenshot of project management features showing summary statistics for participants on a multi-institutional project (organization and contact names were blurred for data privacy).

**Figure 4:**
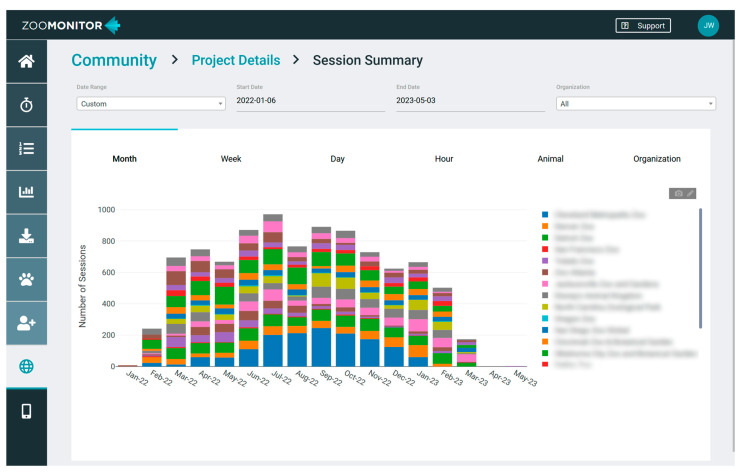
Report from the ZooMonitor Community summarizing the number of sessions by month for a multi-institutional project. Colors indicate participant identity (organization names were blurred for data privacy).

The ZooMonitor Community has multiple features to support PIs in easily managing projects. Any new requests to join the project are organized in a table, where the PI can then review answers for organization-related questions and approve or deny access, which triggers an automated notification email from ZooMonitor to the person requesting access. After participants join the shared project, the PI can track data collection by participants. The PI can view summary statistics for each participant (Figure 3), when participants last uploaded their data, the number of pending sessions for upload (i.e., sessions within a participants organizational account that have not been synced with the shared project database), and track the completion of animal-related and habitat-related questions, ensuring all necessary metadata are provided. In addition, built-in reports summarize data collection efforts by month, week, day, hour, animal, and organization (Figure 4). As needed, built-in reminder emails can be sent from the ZooMonitor Community to nudge participants for different tasks, such as increasing data collection efforts, completing animal-related or habitat-related questions, or syncing participant data with the shared database. Lastly, the PI can export shared data from the ZooMonitor Community project as an Excel spreadsheet for analysis. The data export spreadsheet includes the raw behavior data and answers to organization-, animal-, and habitat-related questions.

## 3. Results and Discussion

**Figure 5:**
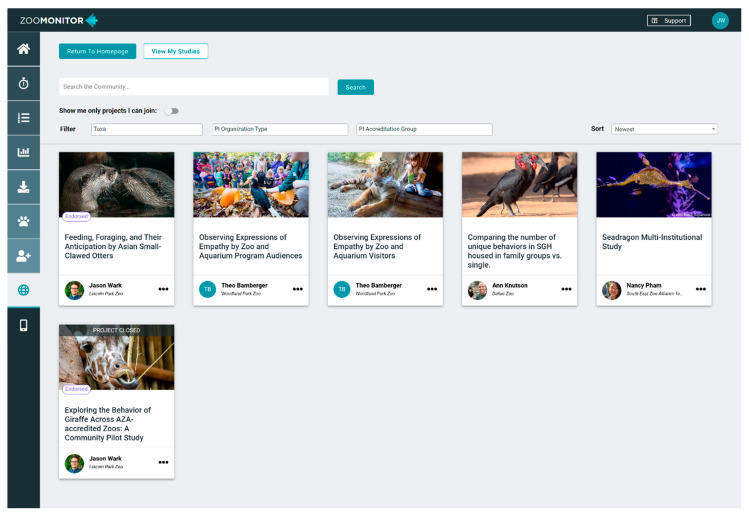
Screenshot of the ZooMonitor Community showing publicly shared projects.

The ZooMonitor Community features were released in 2023 and have been utilized by researchers to address broad, population-level questions on a variety of species (Figure 5). To illustrate the potential value of this new tool for population managers, we highlight several recent case studies, including a project that has been completed (giraffes) and several currently ongoing (otters, seadragons, and hornbills).

### 3.1. ZooMonitor Community Case Studies

#### 3.1.1. Evaluating Housing and Husbandry Factors for Giraffes (*Giraffa camelopardalis*)

Giraffes are one of the most commonly housed megafauna across AZA-accredited zoos, with more than 550 individuals living across more than 100 organizations. Partially owing to that ubiquity, they have been a frequent focus of past multi-institutional research efforts, which often examined their feeding and foraging behaviors. Although giraffes in the wild have been observed to spend the majority of their time browsing [31,32,33], past multi-institutional studies have reported less time spent feeding by giraffes in zoos [34,35,36,37,38]. These studies have also suggested a potential link between the time spent displaying natural foraging behaviors and the prevalence of oral stereotypies, highlighting the importance of considering time budgets for this species [35,37,39]. In addition, giraffes are housed across AZA-accredited zoos in a variety of social group sizes and habitat sizes, but few previous studies have explicitly evaluated these factors. Thus, understanding factors influencing feeding and foraging behaviors and the influence of social group size and habitat size on behavior represent important population-level questions for giraffes.

A multi-institutional study was conducted to provide an overview of giraffe behavior in zoos and identify key factors influencing their behavior. When developing the study, participating organizations were asked to observe a minimum of three individuals from their herds and record a minimum of three to six 10-min observation sessions per week over the course of a year. Some participating zoos chose to observe more than the minimum number of animals from their herds, given their internal desire for additional information that could guide their management decisions. Ultimately, over 9000 observation sessions were recorded, with data from 66 individuals across 18 AZA-accredited zoos, making this study one of the largest giraffe behavior studies ever conducted in zoos. Behaviors recorded for this study included general activities (e.g., inactive, locomotion, browsing), whether the animals were in the sun or shade, and their location within the habitats. As there were over 100 observers recording data for this study, ensuring inter-observer reliability was crucial. A multi-step process was instituted whereby all observers completed an online behavior identification quiz featuring short video snippets of behavior, two video reliability tests, and two in-person reliability tests with a project lead at each organization.

Findings from this study have been shared in several past reports [40,41]. Herein, we highlight several results illustrating the potential value for population managers and the importance of multi-institutional research. As expected from past research, feeding and foraging behaviors were the most common behaviors observed in zoo-housed giraffes and were negatively associated with oral stereotypic behaviors [40]. For both behaviors, there was a large degree of inter-individual variation (e.g., feeding and foraging behavior ranged between 14% and 69% of the time budget across individuals), highlighting the importance of a multi-institutional approach. The researchers were encouraged to note that the mean time giraffes spent feeding during their study was higher than had been reported for past multi-institutional studies, potentially indicating progress across zoos in promoting natural foraging behaviors by giraffes. Through statistical modeling, the influence of housing and husbandry factors on giraffe behavior was identified [41]. For example, contrary to common assumptions, the amount of outdoor space available was not associated with increased locomotion. In addition, large outdoor habitats were negatively associated with browsing behavior, an unexpected finding that may suggest logistical challenges for keepers in servicing large habitats. These types of findings have implications for population managers when describing best practices, such as for Animal Care Manuals.

#### 3.1.2. Assessing Feeding Predictability and Anticipatory Behavior in Asian Small-Clawed Otters (*Aoynx cinereus*)

Asian small-clawed otters have high metabolic rates, potentially twice the rate of comparably sized mammals and relatively higher than other otter species [42]. Based on this and reports that they feed frequently on small prey in the wild [43,44], Asian small-clawed otters are fed multiple small meals per day in managed care. The Otter Animal Care Manual recommends feeding at least three meals per day. In practice, the number of meals may vary across organizations, with some organizations known to feed up to six meals per day (S. Duncan, personal communication). In addition, the scheduling of these meals can also vary across organizations, leading to differences in feeding predictability for otters living in AZA-accredited organizations. Understanding the effects of feeding predictability (or unpredictability) on otter behavior is an important population-level behavior question, as otters are known to display anticipatory behaviors prior to meals (e.g., door banging, screaming), which may signify a welfare concern [45,46,47].

In partnership with the Asian Small-Clawed Otter SSP and with support from the AZA Animal Care and Wellbeing Grant Fund, the first author (J.W.) is leading a multi-institutional study exploring the effects of feeding predictability on anticipatory behaviors by Asian small-clawed otters. Behavior data are being recorded on 18 otters across 9 organizations. As social group size may influence anticipatory behaviors, participating organizations were chosen to provide a balanced sample of pair-housed social groups and larger, family groups. Data collection is being organized in two phases. In the first phase, baseline behavior and space use data are being recorded to examine current behavior patterns by Asian small-clawed otters and evaluate the variation in feeding practices across organizations. In addition to the central focus on feeding predictability, these baseline data are also likely to provide the SSP with valuable insights into social behavior and habitat design. Following this, a second data collection phase will implement automated feeders to experimentally modify feeding predictability, making meals more or less predictable, based on the findings of phase one. The ZooMonitor Community platform has facilitated coordinated data collection across participating organizations and enabled the PI to closely monitor the progress of behavior observations. Similar to the multi-institutional giraffe study, observers are being trained and reliability tested in a three-step process that includes an online behavior identification quiz, video reliability tests, and in-person reliability tests. When completed, this study will provide insights into the behavioral implications of feeding predictability that zoos and aquariums can use to guide how meals are scheduled.

#### 3.1.3. Examining Factors Influencing Reproduction in Common Seadragons (*Phyllopteryx taeniolatus*)

The Association of Zoos and Aquariums (AZA) Marine Fishes Taxon Advisory Group (MFTAG) designated the common seadragon as a TAG-monitored population, coordinated by a designated species champion. Although successful breeding events have occurred at a limited number of aquariums over the past two decades, reproductive success in managed care has remained sporadic. To better understand the behavioral and environmental factors influencing reproduction, important population-level questions for this species, a multi-institutional study was initiated to document courtship behavior across a range of environmental conditions. Both US-based and international EAZA-accredited aquariums were included to ensure a diverse range of housing and care practices were evaluated.

Management of this international collaborative effort was facilitated through the ZooMonitor Community platform, which provided a centralized framework for data collection, training, and communication. Working closely with the species champion and participating aquarists across five aquariums, the team developed a standardized courtship ethogram supported by a catalog of reference videos. These materials—along with observer training modules, inter-observer reliability assessments, and standardized research protocols—were shared within the Community platform to ensure consistent data collection. Flexibility was still able to be integrated into the standardized framework: because individual identification in seadragons becomes increasingly difficult in groups larger than five, institutions were given options to record either the number of animals involved in each behavior or, when feasible, individual-level data. Observers recorded the frequency of courtship behaviors, their spatial distribution within exhibits, and contextual information such as environmental parameters and social groupings across eight consecutive months. Additional flexibility was incorporated to accommodate aquarium-specific needs. For example, recording of additional non-courtship behaviors relevant to husbandry and welfare monitoring was included as desired by participants to capitalize on data collected during observation sessions.

The project will provide valuable insight into seadragon courtship and environmental influences on reproduction. In addition, this study also advanced evidence-based efforts more generally, as it led two aquariums that had not previously used ZooMonitor to adopt the platform for ongoing behavioral monitoring of their seadragon populations and additional species.

#### 3.1.4. Comparing Single and Social Housing for Southern Ground Hornbills (*Bucorvus leadbeateri*)

Southern ground hornbills have been maintained in North American zoological institutions since 1922. Historically, most individuals have been housed singly or in pairs, but recently, several AZA institutions have formed family groups based on field observations of wild southern ground hornbill breeding cooperatively and living in extended family groups [48]. This shift in management presents the first opportunity to systematically evaluate differences in behavior between southern ground hornbills managed in different social groupings, an important and unresolved population-level behavior question.

The goal of this multi-institutional study was to evaluate the behavioral diversity and habitat space use of southern ground hornbills managed singly, in pairs, and in family groups. Because no single institution maintains a sufficient number of birds to allow meaningful statistical comparisons, a collaborative approach was essential. Five zoo partners within AZA, as well as two EAZA institutions, joined the study through the ZooMonitor Community (*n* = 7 institutions). Observers recorded behaviors based on a standardized ethogram that included foraging, vocalizing, self-maintenance, locomotion, social interaction, object manipulation, and exploratory behaviors. Habitat space use was also recorded. Through the Community platform, every participant had access to the same ethogram and data recording parameters to ensure consistency in methodology, and the project manager was able to keep track of the progress of each zoo partner.

This collaboration revealed meaningful data insights, and the commitment of project PIs to communicating the findings with project participants enabled discussions about the management of southern ground hornbills’ social groupings.

## 4. Conclusions

Collaborative behavior research has the potential to rapidly advance our knowledge on how to best care for animals within zoos and aquariums. Furthermore, this research is essential in evaluating dimensions of care that cannot be easily varied within a single organization (e.g., examining habitat size for large megafauna). Even basic, descriptive information from these studies can be a valuable reference for population managers seeking to understand what is normal, and consequently abnormal, behavior. Multi-institutional studies may also serve as an opportunity for benchmarking behavior from which future studies can compare when evaluating long-term progress towards promoting or mitigating specific behaviors, as was possible for giraffes when comparing past multi-institutional studies. The advent of new tools, such as the ZooMonitor Community, has the potential to simplify and streamline multi-institutional data collection, making these studies more approachable than ever. As population management groups serve a critical function within zoo associations in coordinating information exchange and advising on best practices in behavior management, we encourage population managers to prioritize these collaborative studies for the species they manage. Where possible, this could include leading research, either directly or with support from research advisors. Alternatively, population managers could facilitate research by seeking out external collaborators. Taking these steps will also require population managers and other zoo and aquarium professionals to acknowledge the existing gaps in our collective knowledge base and question if current practices are the result of folklore or are evidence-based. Ultimately, building this foundational behavior knowledge will take time, but with the recent growth of behavior monitoring efforts across the community, we believe this is a key moment in the evolution of modern zoos and aquariums.

## Data Availability

The original contributions presented in this study are included in the article. Further inquiries can be directed to the corresponding author(s).

## Abbreviations

The following abbreviations are used in this manuscript:

AZA	Association of Zoos and Aquariums
EAZA	European Association of Zoos and Aquaria
